# IL-33 Enhances the Total Production of IgG, IgG1, and IgG3 in *Angiostrongylus cantonensis*-Infected Mice

**DOI:** 10.3390/tropicalmed9050111

**Published:** 2024-05-12

**Authors:** Po-An Su, Ming-Chieh Ma, Wen-Bin Wu, Jiun-Jr Wang, Wen-Yuan Du

**Affiliations:** 1 Internal Medicine, Infection Department, Chi Mei Hospital, Tainan 71004, Taiwan; d890814@mail.chimei.org.tw; 2 School of Medicine, Fu Jen Catholic University, New Taipei City 242062, Taiwan; 062970@mail.fju.edu.tw (M.-C.M.); wenbin@mail.fju.edu.tw (W.-B.W.); 078625@mail.fju.edu.tw (J.-J.W.)

**Keywords:** *Angiostrongylus cantonensis*, IL-33, eosinophilic meningoencephalitis, angiostrongyliasis

## Abstract

The purpose of this study is to clarify the role of IL-33 in the immune response to angiostrongyliasis, especially in terms of antibody production and isotype switching. In our experiment, C57BL/6 mice were each infected with 35 infectious larvae and were divided into groups that received an intraperitoneal injection of IL-33, anti-IL-33 monoclonal antibody (mAb), or anti-ST2 mAb 3 days post-infection (dpi) and were subsequently administered booster shots at 5-day intervals with the same dose. Serum samples from each group were collected weekly for ELISA assays. The levels of total IgG, IgG1, and IgG3 were significantly increased in *A. cantonensis*-infected mice that were treated with IL-33, and the levels decreased significantly in infected groups treated with anti-IL-33 or anti-ST2 mAb. These results suggest that IL-33 may play a critical role in the pathogenesis of human angiostrongyliasis and could be useful for understanding protective immunity against this parasitic infection.

## 1. Introduction

*Angiostrongylus cantonensis* is a zoonotic public health concern in Taiwan [1], China, Southeast Asia, and the Pacific region [2,3]. *A. cantonensis* is known to use multiple rat species as definitive hosts. The adult worms of this parasite live in the pulmonary arteries and right ventricle of rats, and their eggs hatch in the rats’ lungs. The larvae (L1) then migrate up the trachea and are swallowed and then expelled in the feces of rats. The larvae infect intermediate hosts such as mollusks. Crabs, prawns, and frogs have also been found to be naturally infected and act as paratenic hosts. 

Human infection results from ingesting infective third-stage larvae (L3) that are found in raw or under-cooked intermediate or paratenic hosts. Infection may also be acquired by eating unwashed vegetables. The ingested larvae penetrate the blood vessels of the small intestine and enter the systemic circulation. In humans, the non-permissive host, migration of larvae ceases in the central nervous system. Most of the larvae develop into young adults (L5) but do not fully mature; immature adults generally die shortly after reaching the subarachnoid space. 

In humans, the inflammatory response provoked by the migrating larvae causes the characteristic signs and symptoms of eosinophilic meningitis and eosinophilic meningoencephalitis, such as meningeal irritation with neck rigidity and headache. The immune response of non-permissive hosts is primarily of the Th2 type response, including eosinophilia and increased IgE in the blood and cerebrospinal fluid, and high expression of Th2-type cytokines, especially interleukin (IL)-5, IL-4, IL-13, and IL-33 [4,5,6,7].

The presence of long-lasting antibodies characterizes the humoral immunity generated in response to the infection. The sera contain a mixture of antibodies of different classes (IgG, IgE, IgA, IgM, and IgD) and subclasses (IgG1, IgG2a, etc.), each with its own properties and biological functions [8,9]. Various antigens generated during infection induce antibody class switching via different pathways, facilitated by T helper cells. The CD4+ cells, which recognize the specific antigens, differentiate and secrete cytokines (such as TGF-β, IL-4, etc.) and thereby stimulate class switching in the activated B cells [10]. Specific IgG antibody subclasses in *A. cantonensis*-infected patients have been detected [11]; however, the correlation between the antibody response and cytokines is uncertain in this parasitic disease. 

IL-33, a member of the IL-1 family, is an immunomodulatory cytokine with critical roles in allergic inflammation, fibrosis, tumorigenesis, and homeostasis [12,13,14]. The receptors of IL-33 are IL-1 receptor accessory protein (IL-1RAP) and ST2. ST2 receptors are expressed in many cell types, including eosinophils, mast cells, fibroblasts, and Th2 lymphocytes [15,16].

It is believed that IL-33 is essential for the induction of an effective anti-parasitic immune response. In a murine model of asthma, pre-treatment with soluble ST2 was found to reduce the production of IL-5, IL-4, and IL-13 [17]. IL-33 was previously shown to mediate the expression of IL-5 and IL-13 in angiostrongyliasis [7,18]. Another study showed that blocking IL-33 with an anti-ST2 monoclonal antibody (mAb) could delay the production of IgE [19]. Therefore, the IL-33/ST2 pathway may play an important role in the defense against this parasitic disease.

In this study, for the purpose of understanding the role of IL-33 in the immune response to angiostrongyliasis, especially in antibody production and isotype switching, mice were experimentally infected with larvae of *A. cantonensis* and received injections of IL-33, anti-IL-33 mAb, or anti-ST2 mAb. The total immunoglobulin G, IgG subclass profile, and IgE were assessed using serological examinations. 

## 2. Materials and Methods

### 2.1. Animals 

We maintain *A. cantonensis* in the laboratory by using male Wistar rats as the final host, and *Biomphalaria glabrata,* a hermaphroditic freshwater snail, as the intermediate host. When first infected, each Wistar rat weighed about 150 g. This study used male C57BL/6 mice, whose body weights are 20 g, as non-permissive hosts. 

The rats and mice were labeled pathogen-free, purchased from the Animal Center laboratory at National Taiwan University College of Medicine, and housed following the institutional guidelines. All conducted experiments were approved by the institutional animal ethics committee (approval No. A9846).

All mice survived during the experimental period. The gain in their body weights was similar in infected, normal control, and various treatment groups.

### 2.2. Treatments and Larvae Recovery

The experimental method in this study is a continuation of our previous research [7,19]. We divide mice into the following 8 groups, each with 15 mice. The eight groups included a non-infected group, an *A. cantonensis*-infected group, IL-33-injected groups with or without experimental infection, anti-IL-33 mAb-injected groups with or without experimental infection, and anti-ST2 mAb-injected groups with or without experimental infection. 

The experimentally infected snails were crushed directly with slides and cut into pieces with scissors. Then, we immersed it in distilled water and teased with forceps and needles gently. The infective larvae (L3) were collected under a dissecting microscope. 

We orally infected each mouse in *A. cantonensis*-infected groups with 35 infective larvae (L3) through a tip in 50 µL distilled water. In injected groups, the mice were injected with IL-33, anti-IL33 mAb, or anti-ST2 mAb intraperitoneally at 3 dpi and subsequently injected at 5-day intervals (on days 8, 13, 18, 23, 28, and 33) with the same dose [7,19]. The dose of mouse IL-33 is 1 µg in 0.1 mL of phosphate-buffered saline (PBS) (R&D Systems Inc., Minneapolis, MN, USA). The dose of anti-IL-33 mAb and anti-ST2 mAb is 10 µg in 0.1 mL of PBS (R&D Systems Inc., USA) and 50 µg in 0.1 mL of PBS (R&D Systems Inc., USA), respectively. We collected blood samples from all mice from the tail vein every week (about 0.1 mL of blood from each mouse). The serum samples from each group were collected and pooled for the immunoglobulin assays. 

Three mice from each group were sacrificed by ether anesthesia every week. The brains of infected mice were submerged individually in PBS and gently teased with forceps and needles. The larvae were then collected with a needle and counted under a dissecting microscope.

### 2.3. Immunoglobulin Assays

The levels of total IgG and antibody subclasses, including IgG1, IgG2a, IgG2b, IgG2c, IgG3, and IgE, were determined with the double antibody sandwich ELISA kits (Mouse IgG and subclasses ELISA kit, Innovative Research, Minneapolis, MN, USA) according to the manufacturer’s instructions. In principle, mouse immunoglobulin present in serum reacts with specific antibodies coated on the microtiter wells. After appropriate washing steps, horseradish peroxidase (HRP) labeled polyclonal antibodies are used as the secondary antibody, and chromogenic substrate, 3,3′,5,5′-tetramethylbenzidine (TMB), is used for color development at 450 nm. The quantity of immunoglobulin can be interpolated from the standard curve and corrected for sample dilution. 

### 2.4. Statistical Analysis

In our results, all measured data are presented as mean ± SD and are representative of two independent experiments. Statistical significance was assessed by Student’s *t*-test (two groups) or ANOVA (multiple groups test) to determine significance. *p* value less than 0.01 is considered statistically significant.

## 3. Results

### 3.1. The Levels of Total Immunoglobulin G 

**Figure 1:**
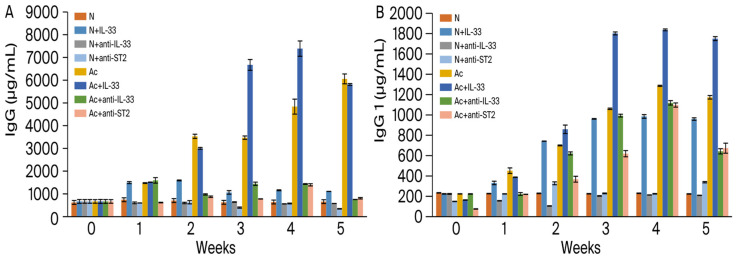
Levels of total IgG (**A**) and IgG1 (**B**) in the sera of mice. N: non-infected mice. Ac: *A. cantonensis*-infected mice. +IL-33: the mice received injections of IL-33. +anti-IL-33 or +anti-ST2: the mice received injections of anti-IL-33 mAb or anti-ST2 mAb. The injections began at 3 dpi and subsequent booster injections were administered at 5-day intervals with the same dose.

The levels of total immunoglobulin G in the sera were analyzed weekly in each group post-infection (Figure 1A). When mice, non-infected or infected, were injected with IL-33, the levels of total IgG significantly increased from the 1st week post-infection (*p* < 0.001). Compared to the non-infected mice, when the infected mice were injected with IL-33, the levels of IgG increased extremely significantly in the 3rd and 4th week post-infection (*p* < 0.001) and reached a peak in the 4th week post-infection. 

In non-infected mice, there was no significant difference between the untreated and anti-IL-33- or anti-ST2 mAb-treated groups. When the infected mice were injected with mAbs, the levels of total IgG decreased extremely significantly in the 2nd week post-infection (*p* < 0.001). 

### 3.2. The Levels of IgG Subclasses

The levels of each IgG subclass in the sera were also analyzed via ELISA and the results are as follows.

IgG1 (Figure 1B): compared to the non-treated and non-infected mice, the levels were increased in all infected and IL-33-treated groups (*p* < 0.001) and reached a peak in the 4th week. Compared to the non-infected mice, when the infected mice were injected with IL-33, the levels of IgG1 increased extremely significantly (*p* < 0.001). Compared to the non-infected mice, when the infected mice were injected with anti-IL-33 mAb or anti-ST2 mAb, the levels of IgG1 decreased highly significantly in the 1st week post-infection (*p* < 0.001).

**Figure 2:**
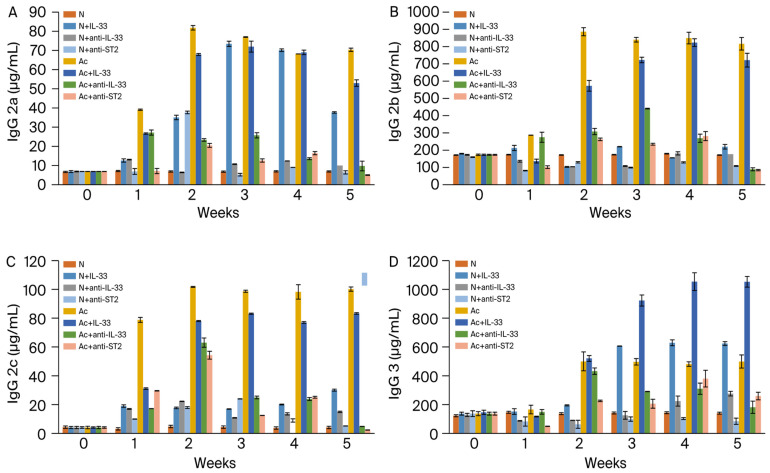
Levels of IgG2a (**A**), IgG2b (**B**), IgG2c (**C**), and IgG3 (**D**) in the sera of mice. N: non-infected mice. Ac: *A. cantonensis*-infected mice. +IL-33: the mice received injections of IL-33. +anti-IL-33 or +anti-ST2: the mice received injections of anti-IL-33 mAb or anti-ST2 mAb. The injections began at 3 dpi and subsequent booster injections were administered at 5-day intervals with the same dose.

IgG2a, IgG2b, and IgG2c (Figure 2A–C): the levels increased in all infected groups with or without IL-33 in the 1st week post-infection (*p* < 0.001). The levels decreased significantly in the infected groups treated with anti-IL-33 or anti-ST2 mAb (*p* < 0.001). 

IgG3 (Figure 2D): the levels increased in all IL-33-treated groups with or without infection in the 2nd week post-infection (*p* < 0.001). The levels decreased significantly in the infected groups treated with anti-IL-33 or anti-ST2 mAb (*p* < 0.001).

### 3.3. The Levels of IgE 

**Figure 3:**
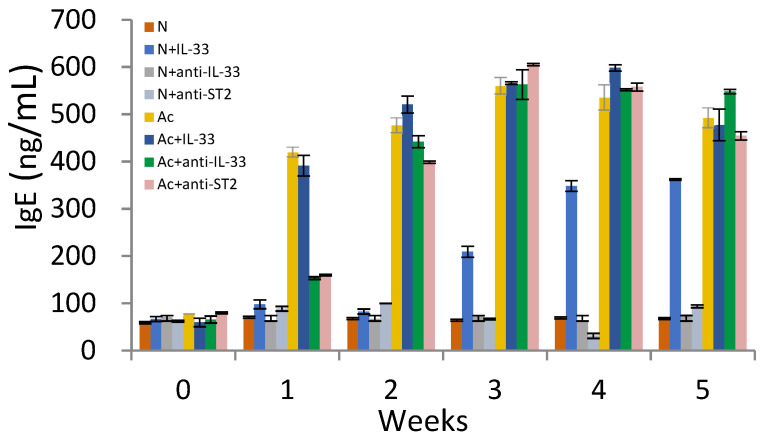
Levels of IgE in the sera of mice. N: non-infected mice. Ac: *A. cantonensis*-infected mice. +IL-33: the mice received injections of IL-33. +anti-IL-33 or +anti-ST2: the mice received injections of anti-IL-33 mAb or anti-ST2 mAb. The injections began at 3 dpi and subsequent booster injections were administered at 5-day intervals with the same dose.

The levels of IgE were increased in the 3rd week in non-infected mice that were injected with IL-33 (Figure 3). When the mice were infected with *A. cantonensis*, the levels of IgE were significantly increased with or without treatment in the 1st week post-infection (*p* < 0.01). 

### 3.4. The Proportions of IgG Subclasses

**Figure 4:**
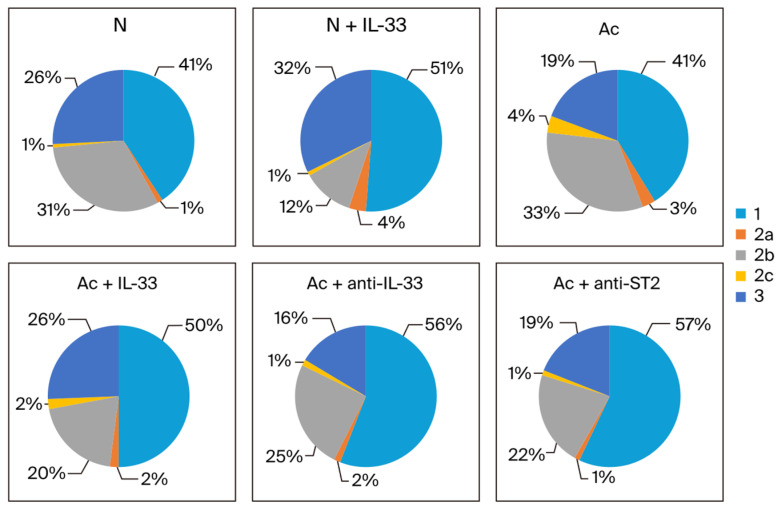
The proportion of each IgG subclass in the sum of IgG1 (1), IgG2 subtypes (2a, 2b, and 2c), and IgG3 (3) in each group. The blood samples were collected at the 3rd week post-infection. N: non-infected mice. Ac: *A. cantonensis*-infected mice. +IL-33: the mice received injections of IL-33. +anti-IL-33 or +anti-ST2: the mice received injections of anti-IL-33 mAb or anti-ST2 mAb. The injections began at 3 dpi and subsequent booster injections were administered at 5-day intervals with the same dose.

The proportion of each subtype in the sum of IgG1, IgG2 subclasses, and IgG3 was analyzed. When non-infected mice were injected with IL-33, the proportion of IgG1 and IgG3 increased in the 3rd week (from 41% to 51% and from 26% to 32%, respectively). Meanwhile, the proportion of IgG2b decreased (from 31% to 12%) (Figure 4).

In the infected groups in the 3rd week post-infection, the proportion of IgG1 increased when the mice were treated (from 41% to 50% when treated with IL-33 and to 56% and 57% when treated with anti-IL-33 and anti-ST2 mAb, respectively). The proportion of IgG2b decreased when the infected mice were treated (from 33% to 20% when treated with IL-33, and to 25% and 22% when treated with anti-IL-33 and anti-ST2 mAb). The proportion of IgG3 was increased when the infected mice were treated with IL-33 (from 19% to 26%) (Figure 4).

**Figure 5:**
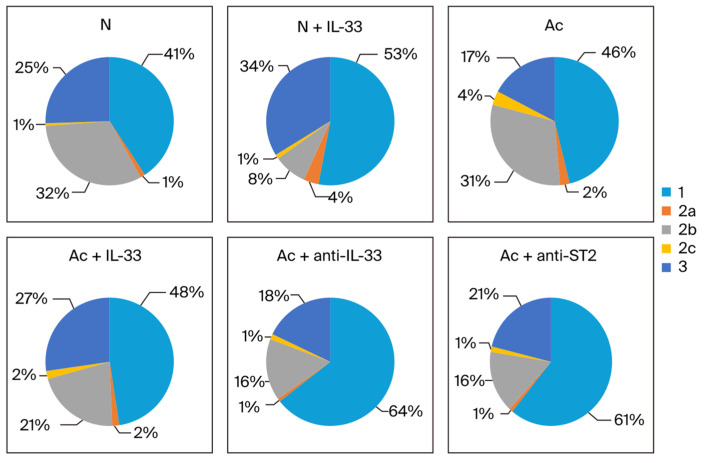
The proportion of each IgG subclass in the sum of IgG1 (1), IgG2 subtypes (2a, 2b, and 2c), and IgG3 (3) in each group. The blood samples were collected at the 4th week post-infection. N: non-infected mice. Ac: *A. cantonensis*-infected mice. +IL-33: the mice received injections of IL-33. +anti-IL-33 or +anti-ST2: the mice received injections of anti-IL-33 mAb or anti-ST2 mAb. The injections began at 3 dpi and subsequent booster injections were administered at 5-day intervals with the same dose.

In the 4th week, when non-infected mice were injected with IL-33, the proportion of IgG1 and IgG3 was increased (from 41% to 53% and from 25% to 34%). In the infected groups, the proportion of IgG1 was increased when the mice were treated with mAbs (from 46% to 64% when treated with anti-IL-33 and to 61% when treated with anti-ST2 mAb). The proportion of IgG2b decreased when the mice were treated (from 31% to 21% when treated with IL-33 and to 16% when treated with anti-IL-33 and anti-ST2 mAb). The proportion of IgG3 was increased when the mice were treated with IL-33 (from 17% to 27%) (Figure 5).

### 3.5. Larva Recovery

In the non-treated group, larva recovery was 13.4 ± 1.95 in the 2nd week post-infection and 11.8 ± 1.64 in the 3rd week post-infection. In the IL-33-treated group, larva recovery decreased to 7.8 ± 0.83 and 6.6 ± 0.54, respectively. The larva recovery was significantly decreased in the IL-33-treated groups (*p* < 0.01). 

There was no significant difference among the groups that were without treatment and treated with anti-IL-33 mAb or with anti-ST2 mAb in the 2nd week and 3rd week post-infection. At the 4th and 5th weeks post-infection, the larva recovery in all groups was low and there was no significant difference among the groups.

## 4. Discussion

Immunoglobulin G, IgG, the principal class of antibody in the blood and extracellular fluid due in part to its longer lifetime, is divided into four subclasses with different heavy chains, and each subclass has distinct properties and effector functions. The main functions of IgG1 and IgG3 are complement activation and the opsonization of pathogens. IgG3 is the strongest activator of complement, followed by IgG1. IgG2 is a weak activator and only activates in the presence of a high antigen concentration [8,10]. 

According to our results, the levels of total IgG, IgG1, and IgG3 were significantly increased in non-infected and *A. cantonensis*-infected mice that were treated with IL-33. The levels were decreased in infected mice that were treated with anti-IL-33 or anti-ST2 mAb. The levels of IgG1 and IgG3 were obviously lower in the anti-ST2 mAb-injected groups in the 3rd and 4th week post-infection. It is necessary to determine the appropriate therapeutic dose for these injected antibodies in the future. However, the IL-33/ST2 pathway may be closely related to the production of total IgG, IgG1, and IgG3. IgG1 and IgG3 have similar properties that activate complement and binding of the Fc receptor [20]. This may be the reason why they are induced together, indicating a shared switch mechanism.

The levels of IgG2a, IgG2b, and IgG2c were significantly increased in infected mice with or without IL-33 injection. However, the levels were significantly decreased when infected mice were treated with anti-IL-33 or anti-ST2 mAb. It seems that IL-33 is necessary for producing these antibody subclasses. 

The ratio changes between antibody classes were taken into account in the 3rd and 4th week post-infection. The proportion of IgG3 was increased in IL-33-treated mice with or without infection. The proportion of IgG2b was decreased when the mice were treated with IL-33 or mAbs. The proportion of IgG1 increased when the infected mice were treated with anti-IL-33 or anti-ST2 mAb. It seems that IL-33 selectively influences the production of each antibody subclass. 

IgE contributes a small but biologically important part of the immune response. The production of IgE occurs in response to helminthic infections and exposure to allergens. In allergic disease, IgE is tightly bound to the surfaces of mast cells and some other cells through the high-affinity IgE receptor FcεRI. The binding of antigen to IgE cross-links the IgE receptors, causing the release of chemical mediators from these cells. However, the mechanism by which IgE fights against *A. cantonensis* is uncertain.

Elevated levels of IL-33 have been found in patients with severe asthma, allergic rhinitis, and conjunctivitis [21,22,23]. IL-33 was shown to enhance the production of IgE in allergic diseases [24]. In this study, IL-33 could enhance the production of IgE in non-infected mice. There were no significant differences in IgE between *A. cantonensis*-infected mice that were treated with or left without IL-33. However, the production of IgE was significantly decreased in both mAb-treated groups in the 1st and 2nd week post-infection. Blocking IL-33 could delay the production of IgE, and this result is consistent with our previous research [19]. 

The isotype switching of antibodies is influenced by several cytokines secreted by T helper cells [25,26]. In human peripheral blood, among B cells stimulated with IL-4 or IL-21, switching is directed to IgG1 and IgG3 [27,28]. It is believed that IFN-γ and IL-4 are antagonistic, and IFN-γ has also been shown to cooperate with IL-6 to induce IgG2 production [29]. In vitro studies have demonstrated that when Th1 IFN-γ is added to the cell culture of murine B cells, IgG2a production is detected [30]. TGF-β may specifically inhibit switching to IgE, rather than IgA [25]. In our study, the levels of IL-4, IFN-γ, and TGF-β were very low, nearly undetectable. The expression levels of these cytokines might need to be determined in cell cultures of lymphocytes co-cultured with antigens of *A. cantonensis* in future work. 

It’s demonstrated that IL-33 is able to modulate intestinal nematode expulsion by inducing the Th2 adaptive response [31,32]. In our study, larva recovery from the infected brains was significantly reduced in IL-33-injected mice in the second and third weeks post-infection. The levels of IgG1 and IgG3 were elevated in these groups. These data suggest that IgG1 and IgG3 could be associated with protective immunity against *A. cantonensis* infection. As for the potential mechanism by which IgG1 and IgG3 limit infection, more experiments are needed.

Taken together, the results of the present study show that exogenous IL-33 enhances the production of total IgG, IgG1, and IgG3 in mice. These antibody levels are significantly increased in *A. cantonensis*-infected mice and may be associated with protective immunity. IL-33 may play a critical role in the pathogenesis of angiostrongyliasis. And the results may be useful when developing treatment strategies against this parasitic disease.

## Data Availability

The original contributions presented in the study are included in the article, further inquiries can be directed to the corresponding author/s.
